# Lung ventilation function characteristics of survivors from severe COVID-19: a prospective study

**DOI:** 10.1186/s13054-020-02992-6

**Published:** 2020-06-06

**Authors:** Xianyong Li, Changsong Wang, Shengjie Kou, Peiyao Luo, Mingyan Zhao, Kaijiang Yu

**Affiliations:** 1 Department of Critical Care Medicine, The First Affiliated Hospital of Harbin Medical University, No.23 Youzheng Street, Harbin, 150001 Heilongjiang Province China; 2 Department of Respiratory Medicine, The First Affiliated Hospital of Harbin Medical University, Harbin, Heilongjiang Province China

**Keywords:** Lung ventilation function, Severe COVID-19, Restriction ventilation dysfunction, Small airway dysfunction

Many studies have been performed on the clinical features of COVID-19 patients [1–3]. There are no reports covering the pulmonary ventilation function of COVID-19 patients, especially severe ones whose lung function may be badly affected. In this study, we described the severe COVID-19 patients’ lung ventilation function.

The First Affiliated Hospital of Harbin Medical University in Heilongjiang Province of China has been designated a treatment center for severe COVID-19 patients in late February. The severe COVID-19 patient in our study refers to the person who has been hospitalized because of COVID-19 with PaO_2_/FiO_2_ ≤ 300 mmHg.

We collected the pulmonary ventilation function variables of survivors from severe COVID-19 who could complete the lung function test near discharge and in quarantine period (2 weeks after discharge); the variables include vital capacity (VC)/predicted value, forced vital capacity (FVC)/predicted value, forced expiratory volume in 1 s (FEV1)/predicted value, forced expiratory volume in 1 s/forced vital capacity (FEV1/FVC), and maximal mid-expiratory flow (MMEF)/predicted value. Before performing a lung function test, we used ultrasonography to exclude the patients who exhibited pleural effusion or pulmonary consolidation.

The SAS v9.1.3 software was used for statistical analysis. The normally distributed quantitative data were described by the mean ± standard deviation (*x̅* ± *s*). A paired *t* test and Wilcoxon rank-sum test were used for comparison of lung function variable values, and the paired chi-square test (McNemar’s test) was used for comparison of lung function type.

We studied 18 patients, including three with a history of smoking and one with a history of tuberculosis. The second test was obtained a mean of 12 days after the first one. The first lung ventilation function tests (the tests near discharge) showed that 8 patients were normal including 2 smokers; 4 patients had restriction ventilation dysfunction including the patient with secondary pulmonary tuberculosis; 2 patients had restriction ventilation dysfunction with small airway dysfunction; 1 patient who also had the smoking history had obstructive ventilation dysfunction; 3 patients had small airway dysfunction. There was no statistical difference in lung function type between the first and the second pulmonary function test (*P* = 0.99) (Table 1).

**Table 1:** Lung ventilation function type and variable values for the 18 severe COVID-19 patients

	First	Second	*P*
Normal	8	8	> 0.05
Obstructive ventilation dysfunction	1	1	
Restriction ventilation dysfunction	4	3	
Small airway dysfunction	3	4	
Restriction ventilation dysfunction with small airway dysfunction	2	2	
VC/predicted value, %	82.8 ± 15.9	88.8 ± 16.5	< 0.01
FVC predicted value, %	84.1 ± 15.4	91.5 ± 17.3	< 0.01
FEV1/predicted value, %	84.5 ± 16.3	89.4 ± 15.7	< 0.01
FEV1/FVC, %	85.2 ± 6.1	80.5 ± 7.0	< 0.05
MMEF/predicted value, %	77.9 ± 27.9	73.6 ± 29.8	> 0.05

Compared with the first test, VC/predicted value, FVC/predicted value, and FEV1/predicted value in the second test were significantly higher (*P* < 0.05), while FEV1/FVC was significantly lower; no significant difference was found in MMEF/predicted value (Table 1).

This report gives a picture of the lung ventilation function of severe COVID-19 patients. The abnormal rate of lung ventilation function was high near discharge, with restriction ventilation dysfunction and small airway dysfunction accounting for 50% of all patients. The result of autopsy obtained from three patients showed interstitial lung inflammation, alveolar inflammatory cell infiltration, mild fibrous hyperplasia, partial alveolar hyaline membrane formation, and alveolar structure destruction [4]; it is suggested that these pathological changes may result in the decrease in lung compliance, which may exhibit restriction ventilation dysfunction in a lung function test. With virus particles observed in distal airway mucosal epithelial through electron microscopy [4], bronchiolitis may exist and result in dysfunction of the small airway. The significant differences of all variable values but MMEF/predicted value suggest that the lung ventilation function is improving except for small airway function, which may require longer-term follow-up.

## Data Availability

The datasets used and analyzed during the current study are available from the corresponding authors on reasonable request.

## Abbreviations

VC: Vital capacity
FVC: Forced vital capacity
FEV1: Forced expiratory volume in 1 s
FEV1/FVC: Forced expiratory volume in 1 s/forced vital capacity
MMEF: Maximal mid-expiratory flow
